# Nutritional status of honey bee (*Apis mellifera* L.) workers across an agricultural land-use gradient

**DOI:** 10.1038/s41598-019-52485-y

**Published:** 2019-11-07

**Authors:** Matthew D. Smart, Clint R. V. Otto, Jonathan G. Lundgren

**Affiliations:** 1 U.S. Geological Survey, Northern Prairie Wildlife Research Center, Jamestown, ND 58401 USA; 2 University of Nebraska, Department of Entomology, Lincoln, NE 68583 USA; 3 Ecdysis Foundation and the Blue Dasher Farm Initiative, 46958 188th St, Estelline, SD 57234 USA

**Keywords:** Ecological modelling, Agroecology

## Abstract

Land use, habitat, and forage quality have emerged as critical factors influencing the health, productivity, and survival of honey bee colonies. However, characterization of the mechanistic relationship between differential land-use conditions and ultimate outcomes for honey bee colonies has been elusive. We assessed the physiological health of individual worker honey bees in colonies stationed across a gradient of agricultural land use to ask whether indicators of nutritional physiology including glycogen, total sugar, lipids, and protein were associated with land-use conditions over the growing season and colony population size the subsequent spring during almond pollination. Across the observed land-use gradient, we found that September lipid levels related to growing-season land use, with honey bees from apiaries surrounded by more favorable land covers such as grassland, pasture, conservation land, and fallow fields having greater lipid reserves. Further, we observed a significant relationship between total protein during September and population size of colonies during almond pollination the following February. We demonstrate and discuss the utility of quantifying nutritional biomarkers to infer land-use quality and predict colony population size.

## Introduction

Managed honey bees are wide-ranging, generalist-foraging, social organisms that primarily rely on environmentally-available resources to support the growth, productivity, and survival of their colonies. While numerous factors (e.g. disease, parasites, pesticide exposure, nutrition, beekeeper management) are involved in the ongoing recent losses sustained by beekeepers^1–4^, habitat and forage quality, and associated nutritional stress, have emerged as critical factors influencing the health trajectory of honey bees and their colonies^5–8^. Because honey bee colonies are required to subsist off the landscape in which they are placed by beekeepers, their interactions with those environments have a largely deterministic effect on colony health outcomes.

In the US, the Northern Great Plains (NGP) region is unique in hosting a substantial proportion (30–40%) of the national pool of commercial honey bee colonies during the growing season (May–October). Access to forage resources growing across the region support population growth and honey production throughout the summer^9–11^. Many of these summering colonies, after overwintering in various locations across the country, go on to pollinate almonds in the Central Valleys of California in early spring. The almond crop, worth over $5 billion USD annually, is completely dependent on insect pollination and covers an area of more than 400,000 hectares^12,13^. Because little to no forage is available to honey bee colonies from October–February, resource (pollen and nectar) availability and land-use conditions across the NGP region during the summer directly impact honey bee colony robustness for almond pollination, setting the stage for successful overwintering and resulting robust, healthy colonies to meet early spring pollination-service demands^9,14^.

Land-use trends over the past two decades in the NGP, however, have led to declines in potential pollinator forage habitat and reduced the suitability of the landscape for supporting honey bee colonies^15–18^. Further, nutritionally-mediated forage quality has been previously demonstrated to differentially impact honey bees and colonies in agroecosystems^7,8^. Given the importance of the region as a core beekeeping locale that services the national crop-pollination industry, it is critical to evaluate the outcomes of such land use in the context of pollinator health. Further, in the NGP and across the US, honey bee colonies are putatively stationed among agroecosystems, so it is important to understand how the nutritional health of honey bee colonies is enhanced or diminished due to surrounding land-use conditions.

We examined the nutritional status of individual honey bees in the context of varying land-use conditions in the NGP to elucidate the nutritionally-mediated impacts of land use on honey bee health. We quantified the protein, lipids, stored carbohydrates (glycogen), and total sugars in samples of adult worker honey bees in the fall, a critical time point for the health of honey bee colonies prior to overwintering. Examining the physiological health of honey bees in autumn provides a window into how over-summering land-use conditions affect honey bee colony health, which ultimately dictates overwintering success^7,14,19^.

The nutritional measures we quantified have been previously used to examine the impacts of pesticides in contaminated pollinator forage near agricultural fields on honey bees^20^. Further, others have demonstrated that such a ‘landscape physiology’ approach is insightful for inferring the impacts of differential land-use conditions on colony and individual bee health outcomes^8^. This study was part of a larger project examining the effects of land use on colony health and pollination-service delivery, wherein land-use conditions were found to influence the population size of colonies for almond pollination^14^. Here, we provide results relating individual bee health to the same land-use conditions. In doing so, we describe the nutritionally-mediated effects of the relationship between land use and differential bee health outcomes. Our predictions for this study were:

1. Nutritional status of individual honey bees would be measurably influenced by surrounding land use.
2. Nutritional status of individual honey bees would be correlated with colony population size and survival the following spring during almond pollination.

## Results

**Figure 1:**
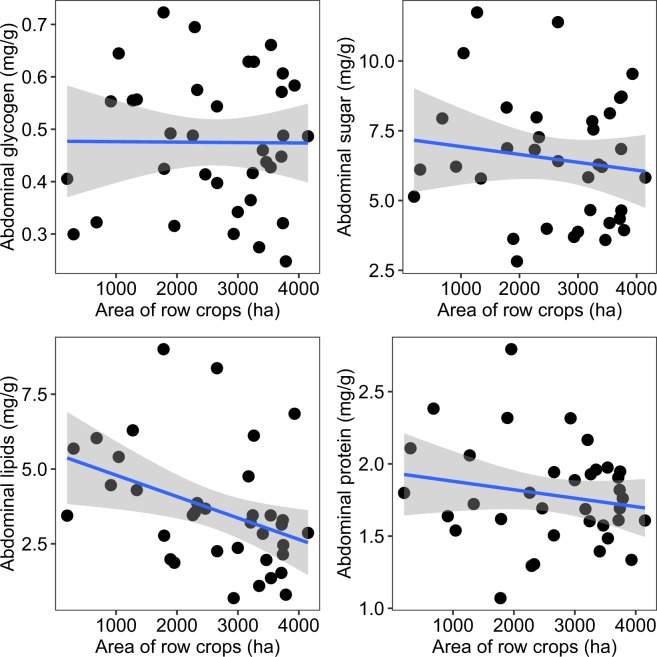
Relationship between the area (hectares, ha) of corn, soy, and small grains (hereafter “row-crop agriculture”) within a 4-km radius (5027 ha) around study apiaries (n = 36) and the levels of four nutritional biomarkers, abdominal glycogen, sugar, lipids, and protein. Each point represents the mean value among all sampled colonies (n = 6) per apiary. Gray area indicates the 95% confidence interval.

We asked whether land use across an agricultural gradient (apiaries surrounded by low to high row-crop presence) influenced nutritional biomarkers in worker honey bees. We observed a significant negative linear relationship between land use and September lipid levels (F_1,34_ = 7.13, r^2^ = 0.17, p = 0.01), wherein lipid levels decreased with greater areas of row crops in the surrounding environment (Fig. 1). The same nutritional indices, and opposite trend, in relation to our other dominant land use category, grassland, are found in SI Fig. 1.

**Figure 2:**
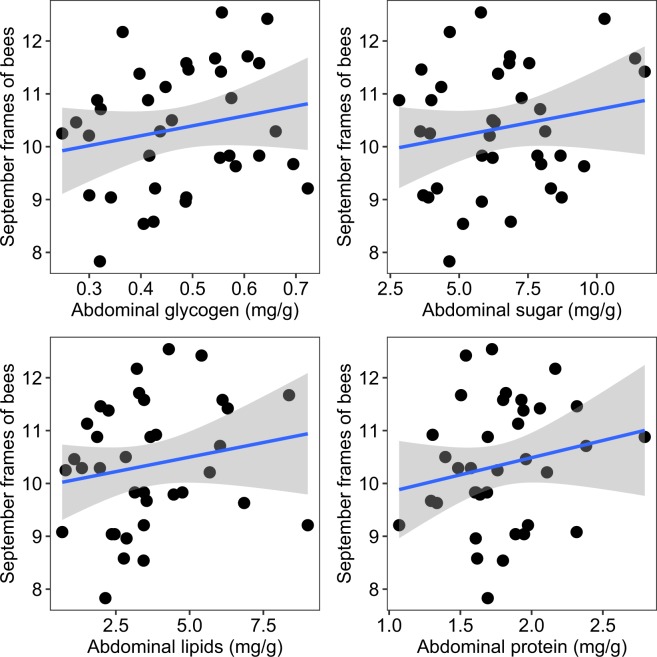
Relationship between levels of four nutritional biomarkers and colony frames of bees in September. Colony population size was calculated as the total number of frames completely covered (on both sides) by adult honey bees. Each point represents the mean value among all sampled colonies (n = 6) per apiary (n = 36). Gray area indicates the 95% confidence interval.

Next, we asked whether nutritional biomarkers in individual bees exhibited a correlation to adult bee population size at the time of sampling (September colony health assessments). None of these observed relationships were statistically significant for our single year of data (Fig. 2).

**Figure 3:**
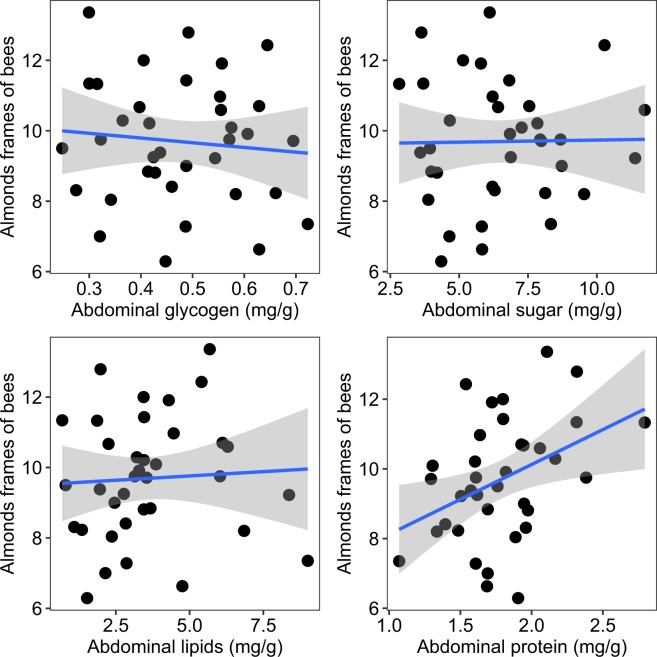
Relationship between the mean level of four nutritional biomarker (quantified in September) and frames of bees during almond pollination. Colony population size was calculated as the total number of frames completely covered (on both sides) by adult honey bees. Each point represents the mean value among all sampled colonies (n = 6) per apiary (n = 36). Gray area indicates the 95% confidence interval.

Insofar as individual bee nutritional biomarkers may relate to colony robustness, we asked whether the measured nutritional health indices related to colony population size during the following February in California almond orchards. Here, protein levels exhibited a positive relationship with colony population size in almonds (F_1,34_ = 6.38, r^2^ = 0.16, p = 0.02), meaning apiaries containing colonies and bees with higher September protein levels were larger for almond pollination (Fig. 3).

Apiary level survival (the proportion of colonies in an apiary surviving from June through to the following February for almond pollination) was not statistically related to any of the quantified potential nutritional biomarkers. (SI Fig. 2).

**Figure 4:**
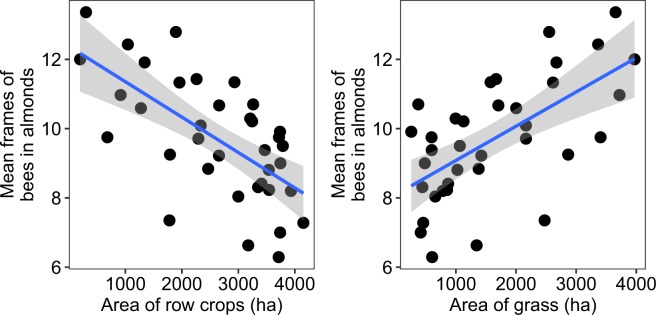
Relationship between the area (hectares, ha) of row crops and grassland within a 4-km radius (5027 ha) around study apiaries during the growing season (May–September, 2016) and the mean apiary-level population size of colonies during almond pollination the following February. Colony population size was calculated as the total number of frames completely covered (on both sides) by adult honey bees. Each point represents the mean value among all sampled colonies (n = 6) per apiary (n = 36). Gray area indicates the 95% confidence interval.

Consistent with our previous work^14^, we found land use across the studied agricultural gradient to be related to colony population size in almond orchards during pollination (Row crops: F_1,34_ = 24.92, r^2^ = 0.42, p < 0.0001; Grassland: F_1,34_ = 21.61, r^2^ = 0.39, p < 0.0001). meaning 42% of the variation in population size among apiaries during almond pollination could be attributed to summer row crop land use conditions, with greater areas of row-crop agriculture (corn, soy, small grains) resulting in smaller population sizes for almond pollination (Fig. 4).

We asked which of the quantified nutritional biomarkers were most strongly related to colony size during almond pollination (Table 1). Models containing September protein levels and lipid levels were the most supported (ΔAICc <2 from top model) for relating fall nutritional biomarkers to colony size during almond pollination, though the 95% CI overlapped zero for fall lipids. These two top models including protein and lipids accounted for nearly 50% of the total weight (*w*) among all models and, further, protein was included in all but the final intercept-only model, highlighting it as a critical fall indicator of future colony strength for almond pollination. Based on the top model, each increase of 0.5 mg/g protein in September resulted in a 1-frame of bees increase in the average colony per apiary the following February, during almond pollination.

**Table 1:** Models relating September nutritional biomarkers to population size (frames of adult honey bees) during almond pollination. Models that were >2 AICc from the intercept-only model were not considered due to a lack of evidentiary support.

Model name	AICc	ΔAICc	*w*	Estimate	±95% C.I.
				Intercept: 6.12	3.19, 9.06
Protein	142.01	0.00	0.24	Protein: 2.00	0.39, 3.62
				Intercept: 4.57	1.04, 8.10
Protein	142.05	0.04	0.23	Protein: 2.41	0.74, 4.09
log(Lipids)				Lipids: 0.73	−0.23, 1.68
				Intercept: 4.16	−0.08, 8.40
Protein	142.77	0.76	0.16	Protein: 2.47	0.71, 4.23
Sugar				Sugar: 0.17	−0.10, 0.45
				Intercept: 3.32	−2.23, 8.88
Protein	143.01	1.00	0.14	Protein: 2.71	0.71, 4.72
Glycogen				Glycogen: 3.21	−2.22, 8.65
				Intercept: 3.40	−2.15, 8.95
Protein	144.40	2.39	0.07	Protein: 2.71	0.70, 4.71
log(Lipids)				Lipids: 0.59	−0.51, 1.68
Glycogen				Glycogen: 1.69	−4.44, 7.81
				Intercept: 3.96	−0.31, 8.22
Protein	144.43	2.42	0.07	Protein: 2.55	0.78, 4.32
log(Lipids)				Lipids: 0.56	−0.61, 1.72
Sugar				Sugar: 0.09	−0.24, 0.41
				Intercept: 2.90	−2.79, 8.59
Protein	144.96	2.95	0.05	Protein: 2.80	0.77, 4.83
Glycogen				Glycogen: 2.07	−4.08, 8.22
Sugar				Sugar: 0.13	−0.18, 0.44
Intercept-only	145.79	3.78	0.03	Intercept: 9.69	9.10, 10.29

ΔAICc represents the difference between AICc values of each model and the top-ranking model; *w* is the AICc model weight. Models are arranged from greatest evidentiary support (lowest AICc, highest *w*) to least support (highest AICc, lowest *w*).

## Discussion

Grassland and semi-natural habitats situated among agricultural lands have been previously shown to positively influence the health, productivity, and survival of honey bees^7,8,14^. Here we tested whether land use acts on colonies within apiaries by improving individual bee health. September lipid levels were not strongly correlated with overwintering survival or colony size in February-blooming almonds. Rather, among the physiological markers we evaluated, September protein levels were most closely indicative of population size the following February, i.e. fall lipids levels reflected growing season land use quality, while protein levels indicated future, post-overwintering colony robustness. This finding corroborates previous research examining the relationship between nutritional health status of honey bees and land-use conditions^7,8,21^.

These findings contribute to our understanding of the measured nutritional biomarkers as they relate to seasonal colony nutritional requirements, population growth, and overwintering physiology of honey bees^22–24^. For example, environmental and colony cues prior to overwintering result in newly-emerging adult (nurse) worker bees shifting to storage of nutritional resources rather than using them for brood-food production, thus triggering their transition to long-lived overwintering bees^25–27^. The shift from short-lived summer bees to long-lived winter bees is specifically accompanied by an increase in hemolymph proteins and lipids, including the storage glycolipoprotein, vitellogenin, allowing winter bees to survive for several months solely on carbohydrates^25,28^. In honey bees, vitellogenin levels are associated with overall health status and longevity^29–31^. Related, the presence of sufficient stored proteins in overwintering honey bees, along with stored colony-level protein (beebread and pollen), allow for protein synthesis and the re-initiation of brood rearing in midwinter^32,33^. This colony population turnover replenishes the workforce and provides colonies with an edge for spring resource foraging and subsequent colony growth and survival^33^, which beekeepers take advantage of when supplying bees for the pollination of February-blooming almonds.

Within the context of our study, these seasonal nutritional and physiological changes in honey bees and colonies have the potential to interact with the environment in concerning ways; honey bees from apiaries surrounded by increasing areas of non-forage habitat over the growing season exhibited reduced lipid stores in the autumn, while a reduced (protein) nutritional state had downstream effects on population size when provisioning pollination services to almonds. Further, interactions between the parasitic *Varroa* mite, residual beekeeper-applied acaricides, and pervasive agrochemical use in agricultural settings characterized by corn, soybean, and small grain crop production have the potential to further alter and reduce the nutritional state of honey bees^20,31,34,35^ We previously modeled the impact of agricultural land use (area of corn, soy, small grains surrounding apiaries), *Varroa* mite levels, disease symptom incidence, and queen events on colony size after overwintering and found land use to be the main driver, with the other variables contributing negatively to the overall effect^14^. Further, high rates of *Varroa* mite infestation have been demonstrated to negate the potential benefits of high quality forage and land use conditions, underscoring the importance of this parasite in influencing colony health and survival outcomes^21^. It is now recognized that *Varroa* mites selectively feed primarily on the fat body; the site of protein synthesis (e.g. vitellogenin) and anti-microbial peptide and detoxification enzyme production, thus contributing to compromised nutritional and immune systems of honey bees^5,36^.

We conducted our study in the NGP, an area supporting nearly 1,000,000 honey bee colonies during the summer. Thus, our study has relevance to meeting national goal established by the Pollinator Health Task Force (2015) of “reducing honey bee colony losses to no more than 15% by 2025”^37^. The NGP landscape, consisting of a mixture of annual row crops intermixed with untilled rangeland and prairies, is representative of much of the Great Plains including the Playa region of the southern plains and the Midwest region of the US. It is unlikely our research findings can be extended to the far western or eastern US where forest is the most abundant natural land-cover. Forests, unlike grasslands, provide pollinator forage during a brief window of time, when woody plants are blooming. Beyond this episodic bloom period, forests are unlikely to produce mass-flowering crops in the understory that are attractive to honey bees. Therefore, our results have relevance to other grassland systems, but not necessarily in areas where grassland is not the dominant native cover.

Given the nature of some pollination contracts, wherein beekeepers receive a differential payment dependent on a minimum number of frames of bees (e.g. 6- or 8-frame minimum) or increased payments based on incrementally larger population sizes^38^, the ability to predict post-overwintering colony strength in the preceding fall has practical management implications for beekeepers. For example, provided a better understanding of how nutritional levels indicate post-overwintering population size, beekeepers may supplement nutritionally-deficient colonies in the fall to enhance the nutrient levels of bees and colonies in their operations and increase their realized income for almond pollination the following spring.

There were limitations inherent to our study that should be addressed going forward before action be taken based on the models presented in this study. First, this experiment was based on a single year of data. Additional years, apiary locations, varying land use conditions, and sampling time points throughout the year would aid in providing a more robust assessment of the interplay between land use, nutrient levels in bees, and related prediction of colony population sizes. Second, we did not remove the alimentary canals of the honey bees analyzed in this study and therefore it should not be assumed that the nutritional values we observed are reflective solely of the bees’ nutrition levels in an absolute sense. Undoubtedly, pollen and nectar contained within the guts of the analyzed bees contributed and elevated our quantified nutrient levels. Finally, and related, while we sampled from the same general location within each colony and during a time of season when colonies were theoretically comprised physiologically of overwintering adult honey bees, we did not control the age of adult bee sampled. To do so was unfeasible given the spatial extent of the study relative to the time and labor that would have been required to rear and mark individual age cohorts of bees within a 7-day window among 3 states, 10 counties, 36 apiaries, and 216 colonies.

Informative next steps for this research would be to determine whether the observed relationships between land use, colony growth, and individual bee nutrition are robust in additional sampling years and across a more diverse suite of land use and climatic conditions. Additionally, it is important to understand whether enhancement of the relevant nutritional biomarkers may be influenced by fall or overwinter nutritional supplementation, and whether such supplemental feeding results in increased colony size during the subsequent February, particularly for honey bee colonies positioned in apiaries surrounded by sub-optimal land use conditions in the summer. While the evidence for supplemental protein to boost colony population size suggests a benefit^39,40^ (but see^41,42^), economic analyses are generally lacking in defining benefits relative to costs. Further, recent work has elucidated the importance of natural over non-pollen based, substitutive protein sources^43^. Also, very high protein diets and those containing elevated protein: carbohydrate ratios have been associated with reduced longevity and other detrimental effects in honey bees^44,45^ and ants^46^. Therefore, careful evaluation of the benefits of such supplementation, optimal timing of feed provisioning, and the type of supplementation for a given beekeeper and his/her operation, should be carried out prior to justifying the costs of product and labor.

Here, and previously^14^, we have demonstrated that land use across an agricultural gradient has substantial impacts on the growth, health, and productivity of commercial honey bee colonies and their ability to deliver ecosystem services. Our work builds upon previous research investigating how ecosystem services provided by migratory species in one part of the country are spatially subsidized by land management activities in another region^47^. Here we show the importance of grasslands such as pasture and the Conservation Reserve Program in supporting almond pollination in California via commercially transported honey bees. Regionally, nationally, and globally, habitat loss and land-use change continue to be driving factors negatively influencing pollinators and other wildlife, so it is critical we characterize how such changes impact outcomes for species such as the economically-important honey bee^16,48,49^. Promoting healthy populations of honey bees by ensuring the availability of adequate habitat, and the forage resources contained therein, contributes to the future health and sustainability of our agricultural system and society.

## Materials and Methods

**Figure 5:**
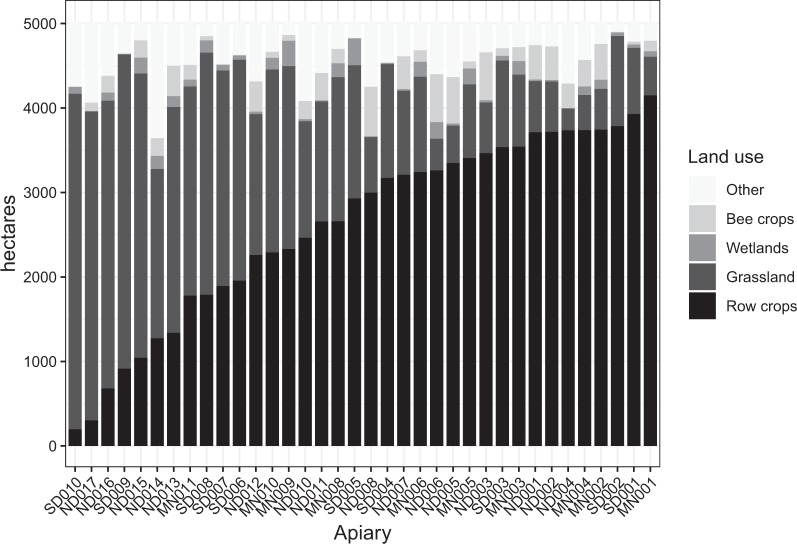
Stacked barplots of land use within a 4-km (5027 hectare) radius of each study apiary in North Dakota (ND), South Dakota (SD), and Minnesota (MN), 2016. The row-crop agriculture category included corn, soybean, and small grain crops (wheat, barley, rye, sorghum, oats, millet). Grassland included grass, pasture, conservation lands, fallow fields, wildflowers, and hay land. Wetlands included both herbaceous and woody wetlands. Bee crops included alfalfa, canola, and sunflower. The “other” category was primarily comprised of open water and developed land.

In June of 2016, six commercially-managed honey bee colonies were randomly selected from each of 36 apiaries situated across an agricultural land use-gradient (Fig. 5) in North Dakota, South Dakota, and Minnesota^14^. Colonies were inspected and their health and colony strength (population size) were assessed. Colonies selected to be included in the experiment were queen-right, contained no less than 5 frames of adult bees, and displayed no overt signs of disease or elevated parasite levels^14^. The collaborating commercial beekeepers (n = 2) managed all of the research colonies in the same manner with respect to timing and treatment of diseases and parasites^14^. Prior to sampling bees in September, colonies were again assessed to ensure a queen-right status (i.e., presence of a queen as verified by visual observation or via presence of eggs and brood) and absence of overt disease symptoms and elevated mite levels to eliminate the confounding factors of queen issues, diseases, and parasites. If a colony was found to have any of these issues, the colony was removed from our sample and another colony within the apiary was randomly selected for sampling. Various colony-level parameters were quantified^14^ including (relevant here) the adult-population size (number of frames of bees) which consisted of estimating the total number of frames per colony with bees covering >90% of each frame^50^.

Approximately 100 adult honey bees were collected in each colony (n = 6 colonies perapiary among 36 apiaries in 2016) during the first week of September from frames containing open brood (larval) cells. We collected adult bees from frames containing open brood to minimize the age variance of honey bees in the sample; generally young, nursing adults are found on those frames (feeding larvae). After collection, samples were immediately placed on dry ice in the field and transported back to the laboratory where they were held at −20 °C until the time of analyses.

The head, thoraces, and legs of individual honey bees were removed, and the remaining carcasses (n = 3 per colony, 18 per apiary), containing intact alimentary canals, were homogenized in 200 µL of phosphate-buffered saline solution (PBS; 0.75 g NaCl, 0.35 KCl, 0.28 g CaCl_2_ in 1 L H_2_O); an additional 800 µL of chloroform:methanol (2:1 by volume) was then added to each sample. The samples were well-mixed in the solvent, and solids were centrifuged into a pellet (21,130 × *g* for 4 min). The solvent was then equally divided among three 1.5-mL microcentrifuge tubes. The first subsample was for total sugar analysis (hot anthrone). The second subsample was for protein analysis (Bradford assay), and the third subsample was for lipid analysis (Vanillin assay). Finally, the pellet of bee tissue was subjected glycogen analysis (hot anthrone).

For total carbohydrates, the homogenized abdomens were diluted in water so that the values fell within the quantifiable range of the test. Fifty µL of the diluted samples was added to 450 µL chloroform:methanol; samples were boiled at 90 °C to evaporate the chloroform:methanol. Anthrone solution (750 mg of anthrone in 1 L H_2_O) was added to each sample (975 µL), and the resulting solution was heated at 90 °C for 15 min. Absorbance was read on 100 µL of the solution at 625 nm.

Proteins were analyzed in 100-µL aliquots of the samples diluted to fall within the quantifiable limits of the test. Each sample was vortexed for 20–30 s and incubated with 150 µL of protein assay dye reagent (Bio-Rad product #5000006, Bio-Rad Laboratories, Hercules, CA) for 5 min. Absorbance was read at 595 nm.

Lipids were quantified using a vanillin assay. All liquid was boiled off the samples, and the remaining sample was diluted in 603 µL of H_2_O and vortex for 15 s. A subsample (50 µL) was heated at 90 °C until all fluid was boiled away. Sulfuric acid (40 µL) was added, and the sample was then heated for 2 min at 90 °C and was finally cooled on an ice bath. Vanillin reagent (975 µL; 600-mg vanillin,100-mL hot water, 400-mL 85% phosphoric acid) was then added to each sample, and these were cooled for 25 min at room temperature. The absorbance was read at 525 nm on 100 µL subsamples.

Glycogen was quantified in the solid pellet from each sample using a hot anthrone test. Anthrone reagent (975 µL) was added to each sample. These were heated to 90 °C for 15 min, and absorbance was read at 630 nm on 100 µL subsamples.

Three standard curves were run on each multi-well plate. Sucrose (#S7903, Sigma, St. Louis, MO, 63103), Bio-Rad protein standard II (#500-0007; Bio-Rad Industries), olive oil (Pompeian Virgin, Pompeian Inc, Baltimore, MD, 21224), and glycogen (SLBX2021; Sigma) were used as the standards for the carbohydrates, proteins, lipids, and glycogen assays, respectively. For the carbohydrate, lipid, and glycogen assays, curves spanning 0, 0.001, 0.01, 0.1, 0.25, 0.5, 1, and 2.5% of the standards were run. The protein curves spanned 0, 12.5, 25, 50, 75, 125, 160, 200 µg/mL. Values from each bee collected in each colony by apiary (n = 18 per apiary) were averaged to determine the mean of each of the nutritional levels per apiary, which were then used for statistical analyses.

We modeled the relationships between land use, individual bee nutrition, colony-population size, and survival using linear regression in R^51^. Normality was tested on all variables using the Shapiro-Wilk normality test. Lipid levels were log-transformed to achieve a normal distribution and were analyzed as such; depictions of lipid levels in figures are based on untransformed data to improve figure interpretability. Attempts to transform apiary survival (as a proportion of surviving colonies) to achieve normality were unsuccessful. As a result, to evaluate the relationship between nutritional biomarkers and survival, we conducted Kendall-Theil nonparametric linear regression on those data. Model selection relating nutritional biomarkers to population size in almonds was carried out using Akaike’s Information Criterion corrected for small sample size (AICc), ranking the multiple competing models relative to AICc values and observing model weights (*w*) and 95% confidence intervals to determine relative significance among models.

## Supplementary information

Supplementary Figures 1 and 2

## Data Availability

The data reported in this article are archived in the U.S. Geological Survey ScienceBase Catalog^52^.
