# Supplementary material for: Nutritional status of honey bee (Apis mellifera L.) workers across an agricultural land-use gradient
Source: Sci Rep. 2019 Nov 7;9:16252. doi: 10.1038/s41598-019-52485-y (PMC6838345; doi:10.1038/s41598-019-52485-y)

Nutritional status of honey bee (*Apis mellifera* L.) workers across an agricultural land-use gradient

Matthew D Smart, Clint RV Otto, Jonathan G Lundgren

Supplementary Information

SI Fig 1. Relationship between the area (hectares, ha) of grassland within a 4-km radius (5027 ha) around study apiaries (n = 36) and the levels of four nutritional biomarkers, abdominal glycogen, sugar, lipids, and protein. Each point represents the mean value among all sampled colonies (n = 6) per apiary. Gray area indicates the 95% confidence interval.

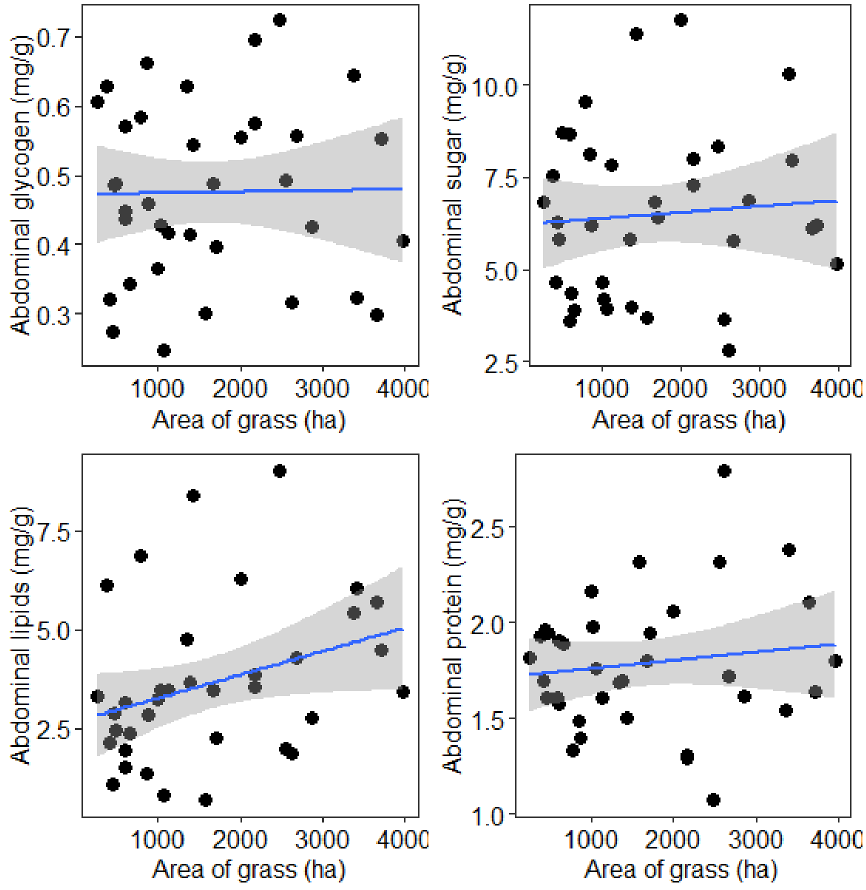

SI Fig 2. Relationship between the mean level of four nutritional biomarker (quantified in September) and the proportion of colonies surviving per apiary from June to the following February for almond pollination. Each point represents the mean value among all sampled colonies (n = 6) per apiary (n = 36).

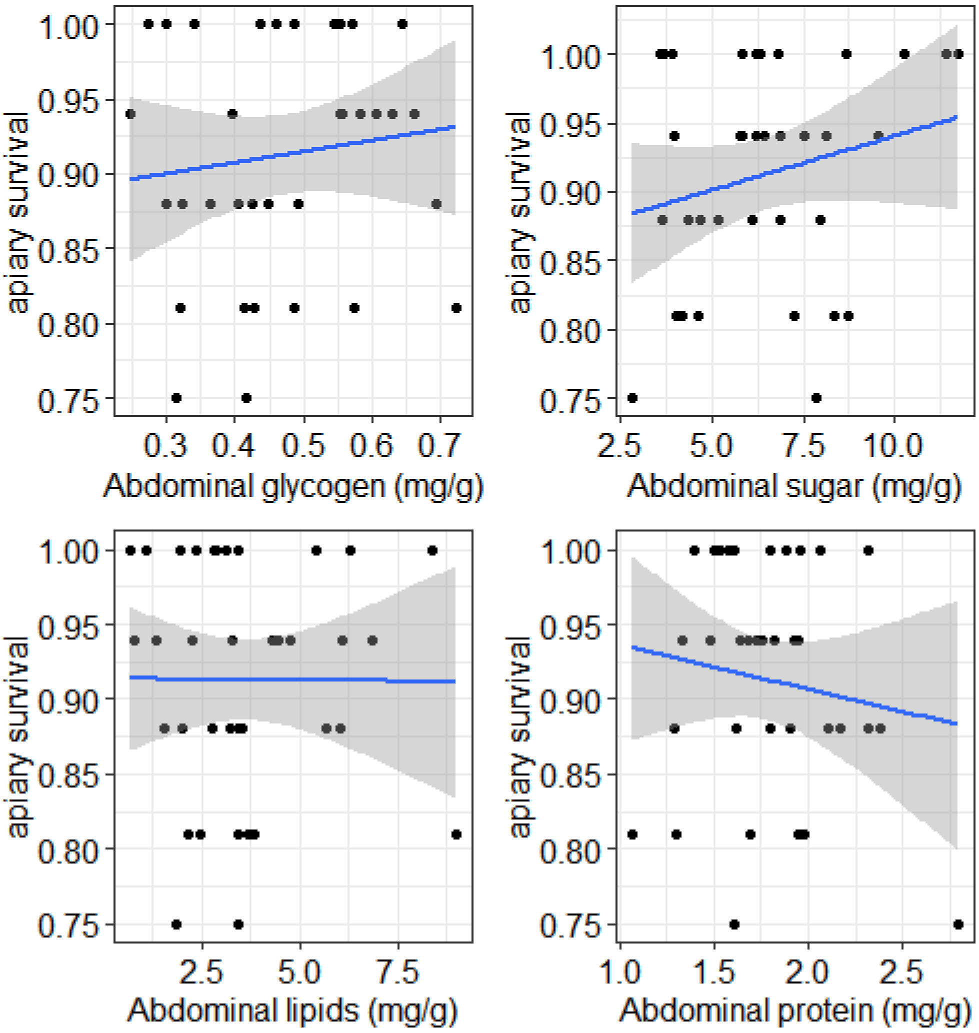

Supplement: Supplementary file 1 — Supplementary Figures 1 and 2 [file 41598_2019_52485_MOESM1_ESM.pdf]
